# Dstac Regulates Excitation-Contraction Coupling in *Drosophila* Body Wall Muscles

**DOI:** 10.3389/fphys.2020.573723

**Published:** 2020-10-06

**Authors:** I-Uen Hsu, Jeremy W. Linsley, Lilly E. Reid, Richard I. Hume, Ari Leflein, John Y. Kuwada

**Affiliations:** ^1^Department of Molecular, Cellular, and Developmental Biology, University of Michigan, Ann Arbor, MI, United States; ^2^Cellular and Molecular Biology Program, University of Michigan, Ann Arbor, MI, United States

**Keywords:** stac adaptor protein, *Drosophila*, muscle, L-type voltage-gated calcium channel, excitation-contraction coupling

## Abstract

Stac3 regulates excitation-contraction coupling (EC coupling) in vertebrate skeletal muscles by regulating the L-type voltage-gated calcium channel (Ca_v_ channel). Recently a *stac*-like gene, *Dstac*, was identified in *Drosophila* and found to be expressed by both a subset of neurons and muscles. Here, we show that Dstac and Dmca1D, the *Drosophila* L-type Ca_v_ channel, are necessary for normal locomotion by larvae. Immunolabeling with specific antibodies against Dstac and Dmca1D found that Dstac and Dmca1D are expressed by larval body-wall muscles. Furthermore, Ca^2+^ imaging of muscles of Dstac and Dmca1D deficient larvae found that Dstac and Dmca1D are required for excitation-contraction coupling. Finally, Dstac appears to be required for normal expression levels of Dmca1D in body-wall muscles. These results suggest that Dstac regulates Dmca1D during EC coupling and thus muscle contraction.

## Introduction

Muscle contractions are initiated by depolarizations of muscle membrane potential due to the release of neurotransmitter at the neuromuscular junction. EC coupling is the process that transduces changes in membrane voltage to increases in cytosolic Ca^2+^ due to the release of Ca^2+^ from the sarcoplasmic reticulum (SR) and subsequently contraction. In vertebrate skeletal muscles EC coupling is thought to be mediated by a direct interaction between the L-type Ca_v_ channel, dihydropyridine receptor (DHPR), which is in the transverse tubule (T tubule) membrane and is the voltage sensor for EC coupling, and the ryanodine receptor (RyR), which is the Ca^2+^ release channel in the SR membrane (Schneider and Chandler, 1973; Rios and Brum, 1987; Tanabe et al., 1987; Block et al., 1988; Takeshima et al., 1989; Adams et al., 1990; Paolini et al., 2004).

Stac3 was identified as a novel adaptor protein that is required for EC coupling in zebrafish skeletal muscle and a missense mutation in *STAC3* is causal for the congenital Native American myopathy (Horstick et al., 2013). Stac3 also regulates EC coupling in murine skeletal muscles (Nelson et al., 2013) and murine muscle development (Ge et al., 2014; Cong et al., 2016). In zebrafish skeletal muscles Stac3 colocalizes with DHPR and RyR and regulates DHPR levels, stability and functionality, including the voltage response of DHPRs but not trafficking of DHPRs (Linsley et al., 2017a, b). Stac3 appears not to be required for normal levels or functionality of RyRs, however.

Recently, a *stac*-like gene, *Dstac*, was identified in *Drosophila* (Hsu et al., 2018). There is a single *stac* gene in *Drosophila* and it is expressed both by muscles and a subset of neurons including in the lateral ventral neurons (LN_V_) that express the neuropeptide, pigment dispersing factor (PDF), in the brain. Genetic manipulation of PDF demonstrated the necessity of PDF for circadian rhythm *in Drosophila* (Shafer and Taghert, 2009). Interestingly, knocking down *Dstac* specifically in PDF neurons disrupted circadian rhythm demonstrating the requirement of Dstac in PDF neurons for normal circadian rhythm (Hsu et al., 2018).

Dstac expression by muscles in *Drosophila* (Hsu et al., 2018) suggests that Dstac might regulate EC coupling in *Drosophila* muscles as does Stac3 in vertebrate skeletal muscles (Horstick et al., 2013; Nelson et al., 2013). As previously mentioned, EC coupling in vertebrate skeletal muscles involves the direct interaction of the L-type Ca_v_ channel, DHPR, in the T tubules with RyR in the SR. In mammalian skeletal muscles DHPR conducts Ca^2+^ from the external solution to the cytosol but this is not required for EC coupling (Dayal et al., 2017). Interestingly in teleost skeletal muscles EC coupling is similarly independent of Ca^2+^ influx from the exterior and DHPR appears to have evolved so that it no longer conducts Ca^2+^ (Schredelseker et al., 2010).

EC coupling in vertebrate cardiac and smooth muscles, however, does require an influx of Ca^2+^ through Ca_v_ channels which initiates Ca^2+^ induced Ca^2+^ release (CICR) from internal Ca^2+^ stores (Bolton et al., 1999). Similarly, EC coupling in invertebrate muscle appears to involve CICR (Györke and Palade, 1992; Maryon et al., 1998; Takekura and Franzini-Armstrong, 2002; Collet, 2009). CICR may also be necessary for EC coupling in *Drosophila* muscles. In *Drosophila* larvae RyR is expressed widely including the body wall muscles and systemic application of ryanodine and a partial loss-of-function *RyR* mutation both decreased locomotion by larvae (Sullivan et al., 2000), which is consistent with the involvement of CICR in body wall muscles for contractions. Furthermore, SERCA, the Ca^2+^-ATPase in the ER/SR that pumps Ca^2+^ from the cytosol into the ER/SR, is expressed by muscles and a dominant heat inducible mutation of *SERCA* paralyzes larvae (Sanyal et al., 2005). Finally, *Dmca1D*, the *Drosophila* L-type Ca_v_ channel is widely expressed (Zheng et al., 1995). *Dmca1D* null embryos exhibit little movement and are larval lethal. Furthermore, pupae of *AR66* partial loss-of-function allele of *Dmca1D* do not eclose (Eberl et al., 1998). Dmca1D in larval muscle conduct voltage-dependent Ca^2+^ currents that are sensitive to dihydropyridines (Ren et al., 1998). These findings suggest that in *Drosophila* larval muscle Ca^2+^ influx via Dmca1D channels might initiate CICR. Here, locomotion analysis, *in vivo* Ca^2+^ imaging, and immunolabeling showed that Dstac and Dmca1D regulate Ca^2+^ transients in muscles and locomotion, and that Dstac is required for normal levels of Dmca1D in the muscles. Our finding suggests that Dstac regulates Dmca1D during the activation of *Drosophila* muscles.

## Materials and Methods

### *Drosophila melanogaster* Strains

All crosses and larvae were kept at 25°C and supplied with food that uses molasses as sugar source (Food R purchased from LabExpress). The number of flies used in crosses was controlled so the vials were not overcrowded with larvae. All experiments used age and size matched larvae. *Dmca1D* knockdown experiments and *in vivo* Ca*^2+^* imaging used 2nd instar larvae of both genders; all the other experiments used both female and male 3rd instar larvae. All experiments were conducted at room temperature (21–23.5°C). *UAS:Dcr-2* was present in all knockdown experiments using RNAi strains except for the TRiP RNAi lines that don’t require Dcr-2. The fly stocks used in this study were: *Mef2:GAL4* (RRID:BDSC_27390), *UAS:Dcr-2* (RRID:BDSC_24651), *UAS:GCaMP6f* (RRID:BDSC_52869), *UAS:mCD4tdtomato* (From Bing Ye), *UAS:Dstac-RNAi* (VDRC 105848), *UAS:Dmca1D-RNAi* (RRID:BDSC_33413), *UAS:Luciferase-RNAi* (RRID:BDSC_31603), *w*^1118^ (RRID:BDSC_3605), *Dstac*^Δ*SH*3^/*CyO* (Hsu, 2020).

### Immunolabeling

3rd instar larvae were fileted in HL3 solution and fixed in 4% paraformaldehyde in PBS. Immunolabeling followed the procedure described previously (Hsu et al., 2018). The primary antibodies used were: chicken anti-Dmca1D (1:20 - 1:100) (Hsu, 2020), rabbit anti-Dstac (1:100 - 1:150) (Hsu et al., 2018), rabbit anti-DsRed (Millipore Sigma Cat # AB356483-25UG), mouse anti-discs large (anti-DLG) (DSHB Cat# 4F3, RRID:AB_528203). Secondary antibodies used were (1:1000): Donkey anti-chicken Alexa Fluor 488 (Jackson ImmunoResearch Labs Cat# 703-545-155, RRID:AB_2340375), Goat Anti-Chicken Alexa Fluor 647 (Abcam Cat # ab150175, RRID:AB_2732800), Donkey anti-chicken Alexa Fluor 633 (Sigma-Aldrich, SAB4600127), Goat anti-rabbit Alexa Fluor 647 (Thermo Fisher Scientific, Cat # A-21245, RRID:AB_2535813), Goat anti-rabbit Alexa Fluor 488 (Thermo Fisher Scientific, Cat # A-11034, RRID: AB_2576217), Goat anti-Mouse Alexa Fluor 568 (Thermo Fisher Scientific, Cat # A-11004, RRID:AB_2534072), Goat anti-Mouse Alexa Fluor Plus 647 (Thermo Fisher Scientific, Cat # A32728, RRID:AB_2633277), Goat anti-Mouse Alexa Fluor 488 (Thermo Fisher Scientific, Cat # A-11001, RRID:AB_2534069). Actin filaments were labeled with Alexa Fluor 647 Phalloidin (Thermo Fisher Scientific Cat # A12379) at 1:1000. Images were acquired with a Leica SP5 and SP8 confocal microscopes using a 100x or 63x oil objective. For comparisons of puncta labeled by anti-Dstac and anti-Dmca1D, images showing only the brightest 50% of the pixels were generated by adjusting the input levels in Photoshop.

### Quantification of Dmca1D Immunostaining of Body-Wall Muscles

Images of muscle 4 from segments A3 to A5 were acquired at 1024 × 1024 pixels as z stacks (5 planes, 0.5 μm/focal plane) with a Leica SP8 confocal microscope with a 100× objective. Confocal settings were kept constant between controls and experimental groups. Images of Dmca1D immunolabeling was quantified in two ways. First, five focal plane images were stacked to a single image using imageJ. ROIs were drawn to encompass the striations labeled with anti-Dmca1D and the fluorescence intensities of the ROIs were measured using imageJ. Second, the anti-Dmca1D fluorescence of pixels in a line of pixels along the longitudinal axis of each muscle that crossed multiple Dmca1D stripes was plotted. The intensities of the pixels along the line minus the background fluorescence of pixels between stripes were analyzed. This method has the advantage of avoiding the selection of ROIs.

### Analysis of Eclosion

Third instar larvae were collected 5 days after the crosses were set. Between 7 and 10 days after the collection of larvae, the number of pupae and adult flies that enclosed were counted. All vials were kept at 25°C.

### Motility Assay

Freely moving 3rd instar larvae were acclimated on a 10 cm 2% agar plate for 1 min and then recorded with a digital camera for 10 s at a frame rate of 7.5 Hz. Each larva was recorded 3 times which constituted a single trial. Larvae that hit the petri dish wall during the 10-s recording were excluded from the analysis. The assay was performed at 23.5°C. Larval movements were tracked by the “multitracker” plugin of imageJ that produced the (x,y) location of each larva in each frame. The distance between frames were calculated from the (x,y) locations and were summed to get the total distance traveled during the 10 s. The controls and experimental groups were coded to blind the genotypes. After completing the assay and analysis, the genotypes were unveiled.

### *In vivo* Ca^2+^ Imaging

Live intact 2nd instar larvae of both genders selectively expressing GCaMP6f in body-wall muscles (*Mef2:GAL4 > UAS:GCaMP6f; UAS:mCD8tdTomato*) were placed into a microfluidics device (Ghannad-Rezaie et al., 2012) and GCaMP fluorescence was observed on a spinning disc confocal imaging system composed of an Olympus IX81 inverted microscope, a CSU-X1 scanner (Yokogawa), an iXon electron multiplying charge-coupled device camera (Andor), and MetaMorph Advanced Imaging acquisition software v.7.7.8.0 (Molecular Devices). Imaging was acquired with a 10X Olympus objective. The larvae were mounted on its side in the chamber in order to image muscle 4, 5, 8, 12, 21 that have some parts that don’t overlap with other muscles. Images were captured every 0.5 sec for 5 min. The *mCD8tdTomato* expressed by *Mef2*:*GAL4* was used to locate the muscles. The larvae that moved a lot were not used. Region of interests (ROI) were drawn over parts of muscles that don’t layer with other muscles and the position of the ROI was re-adjusted manually according to the movements of the samples. Time series analyzer v3 plugin of imageJ was used to measure fluorescence intensity of the ROI. Five frames of GCaMP6f fluorescence before and after the peaks were averaged and used as basal GCaMP6f level (F_basal_) to calculate the fold change of GCaMP6f fluorescence intensity [ΔF/F = (F−F_basal_)/F_basal_]. Prism GraphPad was used to find the peak values. Basal GCaMP6f levels or Ca^2+^ peaks per muscles were averaged as one experiment sample. Both the experiments and analysis were done blind.

### Statistical Analysis

Statistical analyses were performed using Prism GraphPad software. The normality of data distribution was tested by D’Agostino and Pearson test. If the data fit a normal distribution, unpaired *t* test was used. If the data were not normally distributed, the Mann-Whitney test was used. For experiments in which the change in results can be predicted by our hypothesis, one-tailed tests were performed; otherwise two-tailed tests were performed. In all figures, ns, ^∗^, ^∗∗^, ^∗∗∗^, ^****^ represent *P* > 0.05, *P* < 0.05, *P* < 0.01, *P* < 0.001, and *P* < 0.0001. Error bars are standard errors of the mean.

## Results

### Dstac and Dmca1D Are Expressed by Larval Body Wall Muscles

**FIGURE 1:**
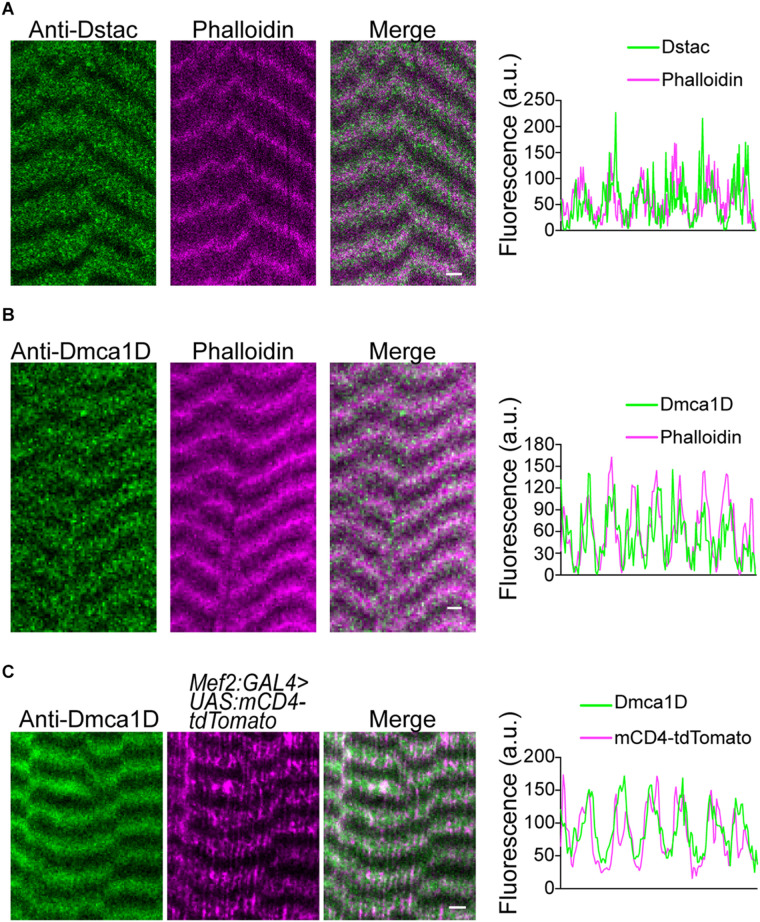
Dstac and Dmca1D are expressed in stripes that are coextensive with T tubules in larval body wall muscles. **(A)** Anti-Dstac/phalloidin double labeling showed that Dstac is expressed in stripes centered on the actin network and Z line of larval muscles. The stripe pattern of the labeling along a muscle by anti-Dstac and phalloidin is quantified to show the extent of overlap of the labeling (right). **(B)** Anti-Dmca1D/phalloidin double labeling showed that Dmca1D is expressed in stripes centered on the actin network and Z line of larval muscles. The stripe pattern of the labeling along a muscle by anti-Dmca1D and phalloidin is quantified to show the overlap of the labeling (right). **(C)** Anti-Dmca1D labeling in *Mef2:GAL4 > UAS:mCD4-tdTomato* larvae showed that Dmca1D is found in the T tubules of larval muscles. The stripe pattern of Dmca1D and CD4-tdTomato along the muscle is quantified to show the overlap of the labeling with the T tubules (right). All images are a single plane. Scale bar, 3 μm.

**FIGURE 2:**
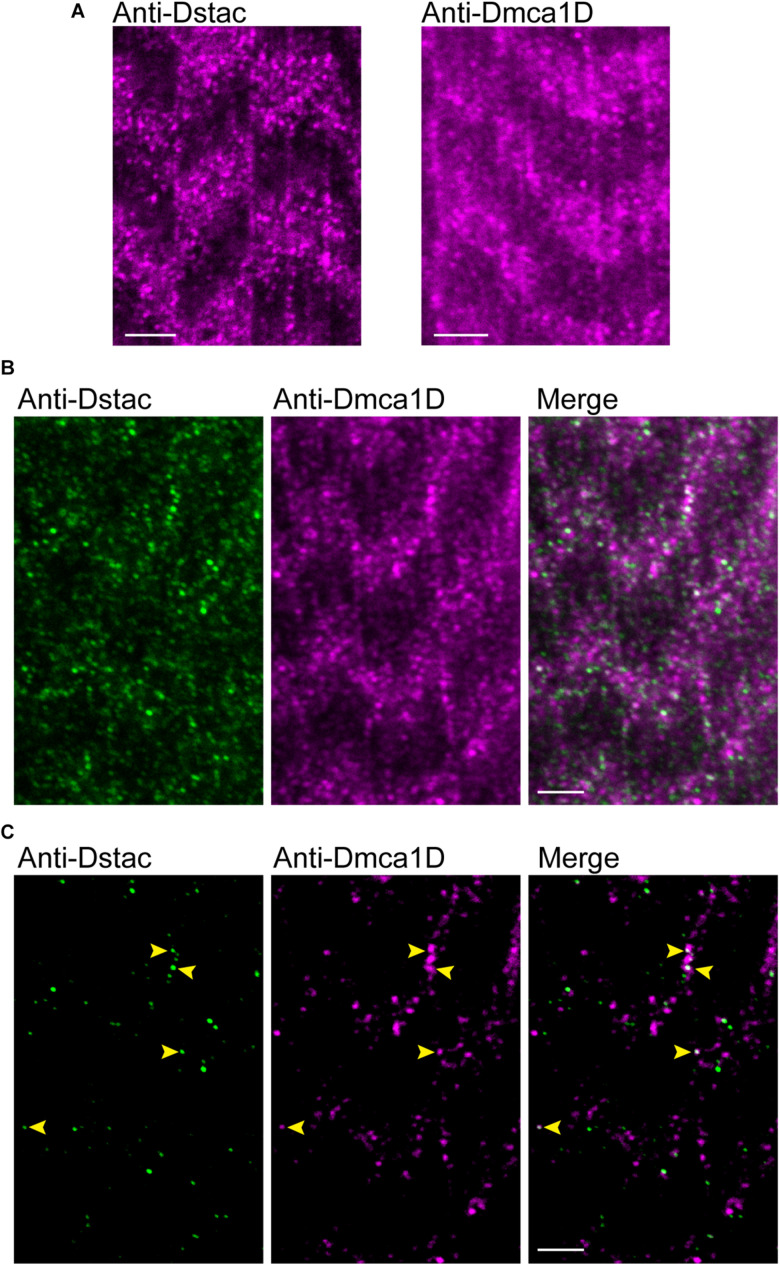
Dstac and Dmca1D are co-expressed in larval muscle stripes. **(A)** Anti-Dstac (left) and anti-Dmca1D (right) labeling of 3rd instar body-wall muscles showed expression of Dstac and Dmca1D in stripes orthogonal to the longitudinal axis of the muscles. The images are a single focal plane. Scale bar, 3 μm. **(B)** Co-immunostaining of 3rd instar larval body wall muscles with anti-Dstac and anti-Dmca1D showed co-expression of Dstac and Dmca1D in the same stripes. The images are a single focal plane. Scale bar, 3 μm. **(C)** Same images as in **(B)** but showing only the puncta made up of the brightest 50% of pixels for easier comparison of the pattern of anti-Dstac and anti-Dmca1D labeling. Arrowheads indicate some puncta that co-labeled with anti-Dstac and anti-Dmca1D. The images are a single focal plane. Scale bar, 3 μm.

In zebrafish skeletal muscles, the L-type calcium channel, DHPR, and cytosolic Stac3 colocalize at specialized junctions of the T tubules and SR (Horstick et al., 2013; Linsley et al., 2017a). To examine the expression pattern of Dstac and Dmca1D within larval body wall muscles, larvae were labeled with anti-Dstac (Hsu et al., 2018) and anti-Dmca1D (Hsu, 2020). The specificity of anti-Dstac was previously demonstrated by Western blot analysis (Hsu et al., 2018) and that of anti-Dmca1D by showing that anti-Dmca1D labeled the CNS in control embryos but no labeling of *Dmca1D* in null embryos (Hsu, 2020). Anti-Dstac and anti-Dmca1D labeled puncta organized as stripes orthogonal to the longitudinal axis of the muscles with the stripes centered on the muscle actin network and sarcomere Z-lines labeled by phalloidin (Figures 1A,B, 2A). Furthermore, co-labeling with anti-Dstac and anti-Dmca1D found that Dstac and Dmca1D localized to the same stripes (Figure 2B) with some puncta co-labeled (arrowheads) for both Dstac and Dmca1D (Figure 2C). Previously, larvae expressing mCD8:GFP that labels the plasma membrane including the T tubules were found in similar stripes in larval muscles (Fujita et al., 2017). In fact, anti-Dmca1D labeled stripes were co-extensive with T tubules expressing mCD4-td-Tomato (Figure 1C), which is consistent with Dmca1D expression in the T tubules. Since anti-Dstac labels the same muscle stripes as anti-Dmca1D, these results are consistent with localization of both Dmca1D and Dstac within the T tubules.

### Larvae With *Dstac* Knocked Down in Body Wall Muscles Exhibited Decreased Locomotion and Failed to Eclose

**FIGURE 3:**
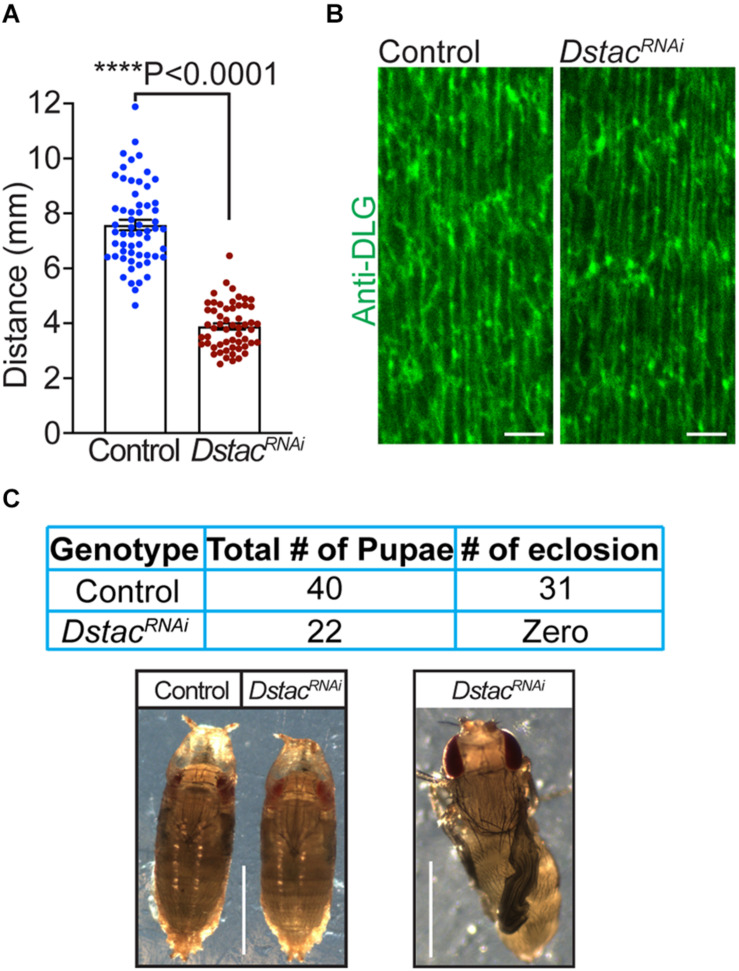
Knockdown of *Dstac* in body wall muscles reduced larval locomotion. **(A)** *Dstac*^*RNAi*^ larvae in which *Dstac* was knocked down in body wall muscles (*Mef2:GAL4 > UAS:Dstac^*RNAi*^*) showed decreased locomotion compared with control *Luciferase*^*RNAi*^ larvae (*Mef2:GAL4 > UAS: Luciferase^*RNAi*^*). Control *n* = 59, *Dstac*^*RNAi*^ *n* = 56. One-tailed, unpaired *t* test. **(B)** Labeling T tubules of body-wall muscles with anti-DLG showed that T tubules of *Dstac*^*RNAi*^ larvae (*Mef2:GAL4 > UAS:Dstac^*RNAi*^*) are similar in morphology and structure to control *Luciferase*^*RNAi*^ larvae (*Mef2:GAL4 > UAS:Luciferase^*RNAi*^*). The images are a single focal plane. Scale bar, 3 μm. **(C)** *Dstac*^*RNAi*^ larvae in which *Dstac* was knocked down in body wall muscles (*Mef2:GAL4 > UAS:Dstac^*RNAi*^*) could develop to mature pharate adults in the pupae but couldn’t eclose. The left lower image shows a control *Luciferase*^*RNAi*^ pupa and a *Dstac*^*RNAi*^ pupa that are similar in morphology. The right lower image shows a pharate adult of *Dstac*^*RNAi*^ that was dissected out from its cocoon in which the wings are not inflated and did not survive. Scale bar, 1 mm. ****P < 0.0001.

Previously we showed that *Dstac* mutant larvae showed decreased locomotion (Hsu, 2020). Since Dstac is expressed both by neurons and muscles (Hsu et al., 2018), the locomotion phenotype could be due to defects in neurons or muscles or both. To see if muscle Dstac is required for normal locomotion, we assayed larvae in which *Dstac* was selectively knocked down in body wall muscles using a muscle driver regulated RNAi line that was previously shown by Western analysis to knockdown *Dstac* selectively in body wall muscles (Hsu et al., 2018). These larvae exhibited decreased locomotion compared with control larvae (Figure 3A). To see if the locomotion defect could be due to defective morphology of muscles in *Dstac*^*RNAi*^ larvae, we labeled muscles with anti-DLG, which recognizes the Disc-large protein, a membrane associated guanylate kinase that is localized to the postsynaptic membrane at the neuromuscular junctions (Lahey et al., 1994) and the longitudinal portions of the T tubules (Fujita et al., 2017). Anti-DLG labeling of the muscles of *Dstac*^*RNAi*^ larvae were comparable with that in control larvae (Figure 3B). This result suggests that knockdown of Dstac in muscles does not lead to any obvious defects in body-wall muscles including the T tubules and thus the decreased locomotion may not be due to any morphological defect in the T tubules. *Dstac*^*RNAi*^ larvae were able to develop to pupae that appeared similar to control pupae, but failed to eclose presumably due to decreased muscle function. The pharate adults released manually from the cocoon by dissection had apparent normal morphology (Figure 3C). Thus, Dstac in body wall muscles is required for normal locomotion.

### Larvae With *Dmca1D* Knocked Down in Body Wall Muscles Exhibited Decreased Locomotion and Muscle Ca^2+^ Transients

**FIGURE 4:**
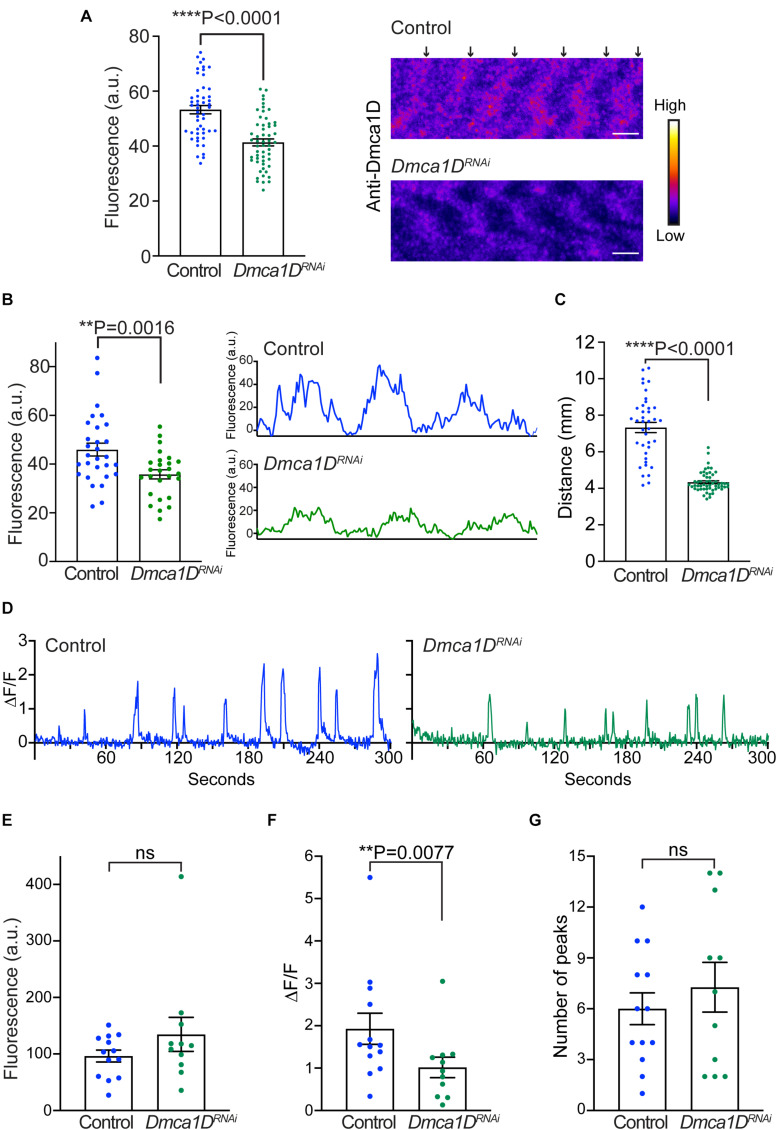
Knockdown of *Dmca1D* selectively in muscles reduced larval locomotion and muscle Ca^2+^ transients. **(A)** Anti-Dmca1D labeling of muscles of larvae with Dmca1D knocked down selectively in body-wall muscles (*Mef2:GAL4 > UAS:Dmca1D^*RNAi*^*) (*n* = 55 stripes from 11 muscles) confirmed that Dmca1D was knocked down compared with control muscles (*Mef2:GAL4 > UAS:Luciferase^*RNAi*^*) (*n* = 50 stripes from 10 muscles). One-tailed, unpaired *t* test. Fluorescence was measured in ROIs that outlined each stripe. Right images are a single focal plane of muscle 4 of a control and a *Dmca1D*^*RNAi*^ larvae. Arrows denote the striations of Dmca1D clusters. Scale bar, 3 μm. **(B)** Same as in **(A)** except the peak fluorescence of stripes was measured. Fluorescence of individual pixels along a longitudinal line that crossed multiple stripes of muscle fibers was measured from *Mef2:GAL4 > UAS:Dmca1D^*RNAi*^* (*n* = 26 stripes from 8 muscles) and control muscles (*Mef2:GAL4 > UAS:Luciferase^*RNAi*^*) (*n* = 29 stripes from 9 muscles). One tailed, unpaired *t* test. Right images are examples of fluorescence measurements along the longitudinal line of wt and control muscles. **(C)** *Dmca1D*^*RNAi*^ larvae in which Dmca1D was knocked down in body-wall muscles (*Mef2:GAL4 > UAS:Dmca1D^*RNAi*^*) showed decreased locomotion compared with control *Luciferase*^*RNAi*^ larvae (*Mef2:GAL4 > UAS:Luciferase^*RNAi*^*). Control *n* = 39, Dmca1D^*RNAi*^ *n* = 54. One-tailed Mann-Whitney test. **(D)** Example of Ca^2+^ transients from a muscle in a control larva (*Mef2:GAL4 > UAS:GCaMP6f; UAS:mCD8tdTomato; UAS:Luciferase^*RNAi*^*) and a *Dmca1D*^*RNAi*^ larva (*Mef2:GAL4 > UAS:GCaMP6f; UAS:mCD8tdTomato; UAS:Dmca1D^*RNAi*^*). **(E)** Expression of GCaMP6f by muscles of *Dmca1D*^*RNAi*^ larvae was comparable to that of control. Mann-Whitney test. **(F)** The peaks of Ca^2+^ transients in the muscles of *Dmca1D*^*RNAi*^ larvae were smaller compared with controls. One-tailed, Mann-Whitney test. **(G)** The number of Ca^2+^ transients over 5 min in *Dmca1D*^*RNAi*^ and control muscles were comparable. Unpaired *t* test. Data in E-G were from 13 muscles of 7 control larvae and from 11 muscles of 6 *Dmca1D*^*RNAi*^ larvae. **P < 0.01 and ****P < 0.0001.

Previously we showed that larvae that were homozygous for the ubiquitous *AR66* partial loss-of-function allele of *Dmca1D* exhibited decreased locomotion (Hsu, 2020). Dmca1D is expressed both by neurons including motor neurons (Hsu, 2020) and muscles and so the mutant phenotype could be due to a defect of *Dmca1D* in either neurons or muscles or both. To see if a muscle deficiency in Dmca1D could lead to decreased locomotion, we selectively knocked down *Dmca1D* in body wall muscles (*Mef2:GAL4 > UAS:Dmca1D^*RNAi*^*). *Dmca1D*^*RNAi*^ larvae developed to the size of 2nd instar larvae and died approximately 10 days after hatching. Anti-Dmca1D labeling in body wall muscles was decreased in 2nd instar *Dmca1D*^*RNAi*^ larvae compared with size-matched control 2nd instar larvae (Figure 4A) suggesting that Dmca1D was indeed knocked down in muscles. We further analyzed the fluorescence levels of pixels along a longitudinal line of each muscle fiber in controls and *Dmca1D*^*RNAi*^ larvae and found that the difference in fluorescence of the peak pixels in the Dmca1D stripe and background fluorescence pixels in non-stripe regions was decreased in *Dmca1D*^*RNAi*^ larvae compared with controls (Figure 4B) confirming that Dmca1D was knocked down. Furthermore, *Dmca1D*^*RNAi*^ larvae showed reduced locomotion compared with size and age matched 2nd instar control larvae (Figure 4C). These results indicate that normal levels of Dmca1D in body-wall muscles are required for normal locomotion.

To see if the decreased locomotion of larvae with a muscle deficiency in Dmca1D was due to a defect in EC coupling, we examined transient increases in cytosolic Ca^2+^ in body wall muscles during locomotion in larvae selectively expressing GCaMP6f in muscles. Ca^2+^ imaging of body-wall muscles was performed in live, intact larvae placed in a microfluidics chamber designed to physically restrain larvae (Ghannad-Rezaie et al., 2012). Under these conditions, Ca^2+^ transient increases were observed within body-wall muscles of control (*Mef2:GAL4 > UAS:GCaMP6f; UAS:mCD8tdTomato; UAS-Luciferase^*RNAi*^*) and *Dmca1D*^*RNAi*^ larvae (*Mef2:GAL4 > UAS:GCaMP6f; UAS:mCD8tdTomato; UAS-Dmca1D^*RNAi*^*) (Figure 4D) that presumably were associated with muscle contractions. The basal GCaMP6f fluorescence levels when larvae were quiescent were similar in *Dmca1D*^*RNAi*^ larvae and control larvae (Figure 4E). Thus, expression of GCaMP6f was unaffected by the knockdown of Dmca1D in body wall muscles. However, the peak of the Ca^2+^ transients recorded from *Dmca1D*^*RNAi*^ larvae was decreased compared with controls (Figure 4F), but the frequency of transients was comparable (Figure 4G). These results showed that the output of the central pattern generator was not dependent on normal Dmca1D in muscles, but normal muscle Ca^2+^ transients during physiological activation of muscles were dependent on Dmca1D. Thus, it appears that EC coupling requires Dmca1D.

### Muscle *Dstac* Deficiency Reduced *Dmca1D* Expression and Ca^2+^ Transients in Muscles During Locomotion

**FIGURE 5:**
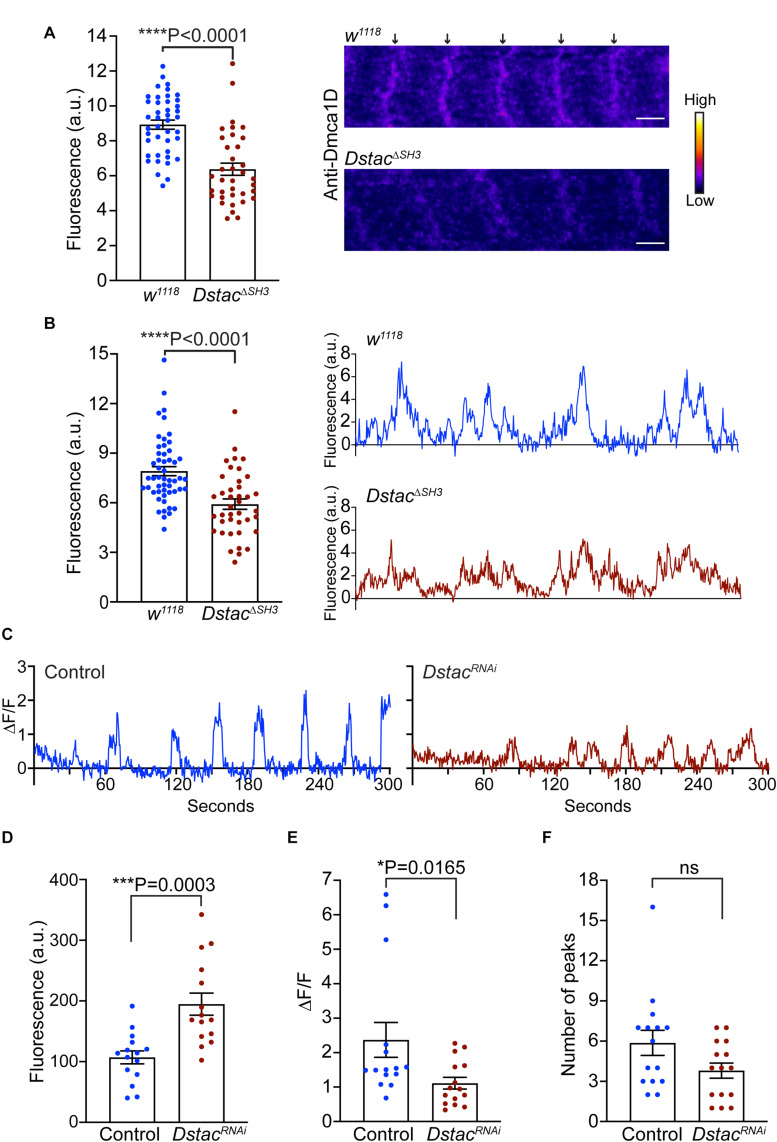
Knockdown of *Dstac* in body-wall muscles reduced Dmca1D expression level and muscle Ca^2+^ transients. **(A)** Anti-Dmca1D labeling of *Dstac*^Δ*SH*3^ larval muscles (*n* = 36 stripes from 12 muscles) showed decreased level of Dmca1D at T tubules compared with wt (*n* = 42 stripes from 14 muscles). One-tailed Mann Whitney test. Right images are a single focal plane of muscle 4 of a wt and a *Dstac*^Δ*SH*3^ larvae. Arrows denote striations of Dmca1D clusters. Scale bar, 3 μm. **(B)** Same as in **(A)** except the peak fluorescence of stripes was measured. Fluorescence of individual pixels along a longitudinal line that crossed multiple stripes of muscle fibers was measured from *Dstac*^Δ*SH*3^ (*n* = 40 stripes from 13 muscles) and wt (*n* = 52 stripes from 14 muscles). One-tailed Mann Whitney test. Right images are examples of fluorescence measurements along the longitudinal line of wt and *Dstac*^Δ*SH*3^ muscles. **(C)** Example of Ca^2+^ transients from a muscle in a control larva (*Mef2:GAL4 > UAS:GCaMP6f; UAS:mCD8tdTomato; UAS:Luciferase^*RNAi*^*) and a *Dstac*^*RNAi*^ larva (*Mef2:GAL4 > UAS:GCaMP6f; UAS:mCD8tdTomato; UAS:Dstac^*RNAi*^*). **(D)** The level of GCaMP6f fluorescence in muscles of *Dstac*^*RNAi*^ larvae was higher than that of control. Unpaired *t* test. **(E)** The peaks of Ca^2+^ transients in the muscles of *Dstac*^*RNAi*^ larvae were smaller compared with controls. One-tailed Mann-Whitney test. **(F)** The number of Ca^2+^ transients over 5 min in *Dstac*^*RNAi*^ and control muscles were comparable. Mann-Whitney test. Data in D-F were from 15 muscles of 5 control larvae and from 15 muscles of 5 *Dstac*^*RNAi*^ larvae. *P < 0.05, ***P < 0.001, and ****P < 0.0001.

Since both Dmca1D and Dstac regulate larval locomotion and Dmca1D regulates Ca^2+^ transients during EC coupling, we asked if Dstac regulates Dmca1D. Immunolabeling the body wall muscles of wt and *Dstac*^Δ*SH*3^ mutant larvae (Hsu, 2020) with anti-Dmca1D showed that Dmca1D expression appeared to be reduced in *Dstac*^Δ*SH*3^ compared with wt larvae (Figure 5A). We further analyzed the fluorescence levels of pixels along a longitudinal line of anti-Dmca1D labeled muscle fibers in controls and *Dstac*^Δ*SH*3^ larvae and found that the difference in fluorescence of the peak pixels in the Dmca1D stripes and background fluorescence pixels in non-stripe regions was decreased in *Dstac*^Δ*SH*3^ larvae compared with controls (Figure 5B) confirming that Dmca1D was diminished in *Dstac*^Δ*SH*3^ larval muscles. The finding that there was no obvious defect in T tubules when Dstac is knocked down (Figure 3B) argues against the possibility that the decreased Dmca1D in *Dstac* mutants might be a by-product of a defect in the morphology/organization of the T tubules. Furthermore, although the intensity of anti-Dmca1D was reduced in *Dstac*^Δ*SH*3^ mutants, the pattern of labeling was not, which is consistent with intact T tubules in mutants. This finding is consistent with the earlier finding that DHPR levels of skeletal muscles were reduced in mouse *stac3* knockout myotubes (Polster et al., 2016) and *stac3* null zebrafish (Linsley et al., 2017a). Thus, the Dmca1D immunolabeling of muscles in wt and *Dstac*^Δ*SH*3^ mutant larvae is consistent with the necessity of Dstac for normal expression of Dmca1D in muscles.

The decreased Dmca1D in body wall muscles predicts that Ca^2+^ transients should also be reduced when Dstac is knocked down in muscles (*Mef2:GAL4 > UAS:GCaMP6f; UAS:mCD8tdTomato; UAS-Dstac^*RNAi*^*). As before Ca^2+^ transients were assayed in larvae selectively expressing GCaMP6f in body wall muscles (Figure 5C). The basal GCamP6f level when muscles were quiescent was higher in *Dstac*^*RNAi*^ larvae compared with control larvae (Figure 5D), but peak Ca^2+^ transients were decreased (Figure 5E) but not their frequency (Figure 5F). Thus, Dstac appears to regulate Dmca1D to mediate normal Ca^2+^ transients during EC coupling.

## Discussion

In invertebrates, CICR appears to be important for muscle contraction (Györke and Palade, 1992; Collet, 2009). We found that *Drosophila* larvae in which *Dmca1D* was selectively knocked down in body wall muscles exhibited decreased muscle Ca^2+^ transients and locomotion. Thus, our results are consistent with the possibility that Ca^2+^ influx via Dmca1D might initiate CICR that leads to Ca^2+^ transients during EC coupling and thus muscle contraction.

In vertebrate skeletal muscles unlike in invertebrate muscles, EC coupling is independent of an influx of Ca^2+^ from calcium channels but rather involves direct interaction of DHPR, the voltage dependent L-type calcium channel in the T tubules, and the RyR Ca^2+^ release channel in the SR (Paolini et al., 2004; Dayal et al., 2017). Stac3 is a key regulator of EC coupling in vertebrate skeletal muscles that regulates the stability and voltage-response of DHPR in T tubules (Linsley et al., 2017a). Despite the differences in EC coupling of vertebrate and invertebrate muscles, the results in this study suggest that Dstac plays a conserved role as vertebrate Stac3 in regulating EC coupling. First, Dstac and Dmca1D are localized to the same stripes of the body wall muscles, which may also correspond with the T tubule network. Second, knockdown of Dstac in body-wall muscles reduced larval locomotion just as when Dmca1D is knocked down. Third, deficiency of Dstac in muscles decreased Ca^2+^ transients in body wall muscles during locomotion similar to that seen when Dmca1D is knocked down. Thus, Dstac is required for normal EC coupling. It appears that stac proteins regulate excitation-contraction coupling in both vertebrate skeletal muscles and invertebrate muscles due to their regulation of L-type calcium channels. In this regard, vertebrate cardiac myocytes that utilize CICR express low basal levels of Stac2, another stac protein (Niu et al., 2018) suggesting the possibility that stac proteins regulate excitation-contraction coupling in vertebrate cardiac muscles as well.

Our finding that the levels of Dmca1D in body wall muscles appeared to be decreased in *Dstac* mutants suggests that Dstac regulates Dmca1D. This finding corresponds with the regulation by Stac3 of the stability and thus the level of DHPRs in zebrafish skeletal muscles (Linsley et al., 2017a). Thus, it is possible that Dstac might regulate the stability of Dmca1D. Experiments such as live imaging of a fusion of Dmca1D with a photoconvertible protein will be needed to assay whether the stability of Dmca1D is regulated by Dstac. Stac3 also regulates the voltage response of DHPRs in zebrafish skeletal muscles (Linsley et al., 2017a). Whether Dstac also regulates the voltage response of Dmca1D await to be examined by voltage clamp experiments. In this regard, voltage-clamp analysis of L-type currents in motor neurons that also express Dstac and Dmca1D found that Dstac was required for normal voltage responses of Dmca1D channels (Hsu, 2020) suggesting that this might also be the case in body wall muscles.

Besides the Src Homology 3 (SH3) and cysteine-rich domain (CRD) that define the Stac proteins, Dstac has a putative BAR domain as do the other invertebrate Stac proteins but not the vertebrate Stac proteins (Hsu et al., 2018). The function of the putative BAR domain of Dstac is unknown. Amphiphysin, a protein containing a SH3 and a BAR domain but not a CRD domain, was found to regulate the development and organization of T tubules and thereby EC coupling (Razzaq et al., 2001). This result is consistent with a role for BAR domains for mediating membrane curvature (Salzer et al., 2017). However, what role the BAR domain of Dstac might play is unclear, but we did not detect any obvious defect in the T tubules of *Dstac*^*RNAi*^ larvae. This finding appears to be consistent with no role of the Dstac BAR domain for the formation of T tubules. However, Dstac is alternatively spliced and *Dstac*^*RNAi*^ targeted the linker sequence between the CRD and SH3 domains that is downstream of the BAR domain (Hsu et al., 2018). In fact, there are 13 transcripts containing a BAR domain and only 3 of these would have been targeted by the RNAi. Thus, any role of Dstac for the formation of T tubules will require further analysis.

Our results showed that *Dstac*^*RNAi*^ larvae exhibited higher basal Ca^2+^ levels during the quiescent stage of locomotion. Control and *Dstac*^*RNAi*^ larvae carried the same number of *GAL4* and *UAS* elements so this difference may not be due to differences in the expression level of GCaMP6f. The increased cytosolic Ca^2+^ levels in *Dstac*^*RNAi*^ muscles could be explained by a decreased sequestration and/or storage of Ca^2+^ within the SR or through an increase in steady-state SR Ca^2+^ leak. However, in *Dmca1D*^*RNAi*^ larvae, the elevated cytosolic Ca^2+^ when muscles were quiescent was not observed as it was in *Dstac*^*RNAi*^ larvae. These results are consistent with the possibility that Dstac might regulate some Dmca1D-independent mechanisms to maintain cytosolic Ca^2+^ levels.

Electrophysiological analysis showed that *Drosophila* larval body-wall muscles express not only the L-type channel, *Dmca1D*, but also the T-type channel, *Dmca1G* (Gielow et al., 1995). Interestingly, vertebrate Stac1 was found to form a complex with a mammalian T-type Ca_v_ channel, Ca_v_3.2, and is required for Ca_v_3.2 expression at plasma membrane (Rzhepetskyy et al., 2016). It would be interesting to examine if Dstac also regulates Dmca1G as suggested by the Stac1/T-type Ca_V_ interactions.

## Data Availability Statement

The raw data supporting the conclusions of this article will be made available by the authors, without undue reservation.

## Author Contributions

I-UH, JL, and JK contributed to the conceptualization. I-UH and JK contributed to the methodology. I-UH contributed to the formal analysis. I-UH, JL, LR, AL, and JK contributed to the investigation. RH and JK contributed to the resources. I-UH and JK contributed to the writing of the original draft. JK contributed to the supervision. I-UH, JL, and JK contributed to the funding acquisition. All authors contributed to the article and approved the submitted version.

## Conflict of Interest

The authors declare that the research was conducted in the absence of any commercial or financial relationships that could be construed as a potential conflict of interest.
